# Supplementary material for: Characterization of PEBP-like Genes and Function of Capebp1 and Capebp5 in Fruiting Body Regeneration in Cyclocybe aegerita
Source: J Fungi (Basel). 2024 Jul 31;10(8):537. doi: 10.3390/jof10080537 (PMC11355433; doi:10.3390/jof10080537)
Supplement: Supplementary file 1 [file jof-10-00537-s001.zip › File S1.pdf]

Supplementary Table S1. Primers used in this study.

Primer	Sequence (5′ to 3′)	Description
capebp1F	TAACGAATAATAGCCGATATCATGCTCGTCTATGCTCAAGACACA	Amplification for the whole length of *Capebp1*
capebp1R	CCGGTCGGCATCTACGATATCCTAAGGTGGGTCGGGTCCA	
capebp3F	TAACGAATAATAGCCGATATCATGCGTTTCTTCGTATCCGC	Amplification for the whole length of *Capebp3*
capebp3R	CCGGTCGGCATCTACGATATCCTAGTAATCCGGCTCCACCAAC	
capebp4F	TAACGAATAATAGCCGATATCATGCGCATCCTCTTATCCACTAT	Amplification for the whole length of *Capebp4*
capebp4R	CCGGTCGGCATCTACGATATCCTATTCACTCCCCATCACCATGA	
capebp5F	AACGAATAATAGCCGATATCATGCCTAGTTCGTACCCCGAA	Amplification for the whole length of *Capebp5*
capebp5R	CCGGTCGGCATCTACGATATCTCACGCTCCAGGCAGGACT	
capebp1RiF	TAACGAATAATAGCCGATATCGGTAATGATACGGCGTAAGGGC	Amplification for partially reversed sequence of *Capebp1*
capebp1RiR	CTCTCGTTTACGGCGGATATCCAAGACAGGGCCGAAAGCT	
capebp5RiF	TAACGAATAATAGCCGATATCCAGGAAGTGGCGAACTTGAGTG	Amplification for partially reversed sequence of *Capebp5*
capebp5RiR	CTCTCGTTTACGGCGGATATCGAGCATCGCTACCTTGAGCCT	
19ha3	TCACCGTAACGAATAATAGCC	Verification of transformants
19ha4	CCCTTATCTGGGAACTACTCAC	
gpd qF	AGGCTGTCGGCAAGGTTATC	Detection for *gpd* expression level
gpd qR	TGCGGTGTGACCAATGAAG	
pebp1qF	TGGATTGCTGAAAGATGTCC	Detection for *Capebp1* expression level
pebp1qR	TTGCTCGTGAGTATGCGTC	
pebp3qF	TTATGTGTCGTCAGGCGTTC	Detection for *Capebp3* expression level
pebp3qR	TCTCCGAGGTTGGTCTTGAG	
pebp4qF	GAGACTCGTTTCTGGGCAAG	Detection for *Capebp4* expression level
pebp4qR	GAGGGATTGAATGGTATCGG	
pebp5qF	TTCTACAACAGGACTCCAGCC	Detection for *Capebp5* expression level
pebp5qR	TTTCCAGCAATAGGGTCTCC	

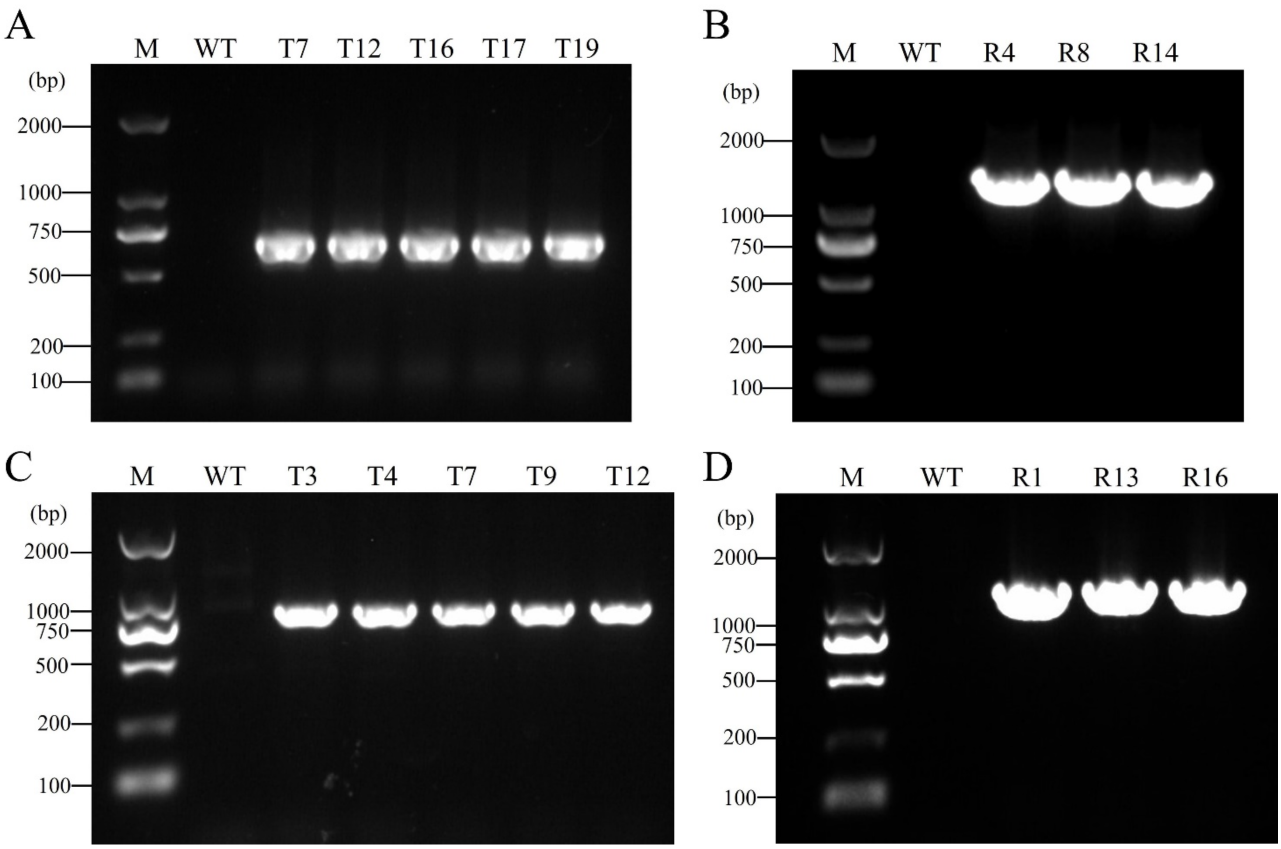

Supplementary Figure S1. Transformants were confirmed by PCR. **(A)** The verification of *Capebp1* overexpression transformants T7, T12, T16, T17 and T19. **(B)** The verification of *Capebp1* RNAi transformants R4, R8 and R14. **(C)** The verification of *Capebp5* overexpression transformants T3, T4, T7, T9 and T12. **(D)** The verification of *Capebp5* RNAi transformants R1, R13 and R16. WT: the wild type.
