# Supplementary material for: Characterization of PEBP-like Genes and Function of Capebp1 and Capebp5 in Fruiting Body Regeneration in Cyclocybe aegerita
Source: J Fungi (Basel). 2024 Jul 31;10(8):537. doi: 10.3390/jof10080537 (PMC11355433; doi:10.3390/jof10080537)
Supplement: Supplementary file 1 [file jof-10-00537-s001.zip › File S2.pdf]

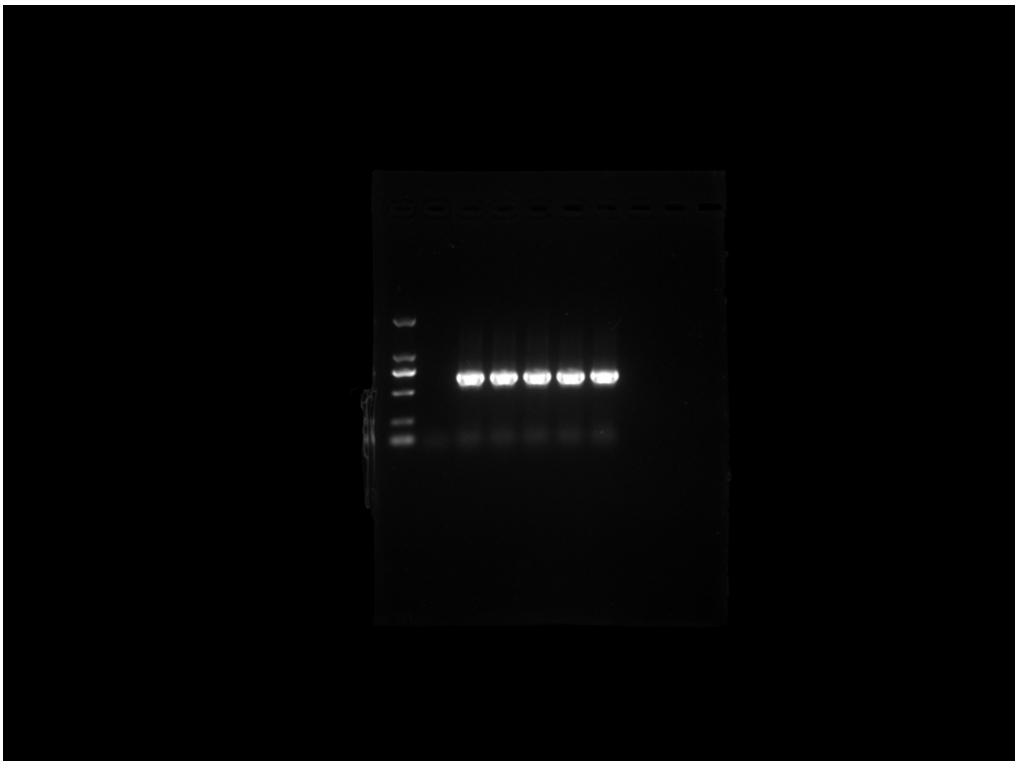

A: The raw gel of Supplementary Figure S1. A

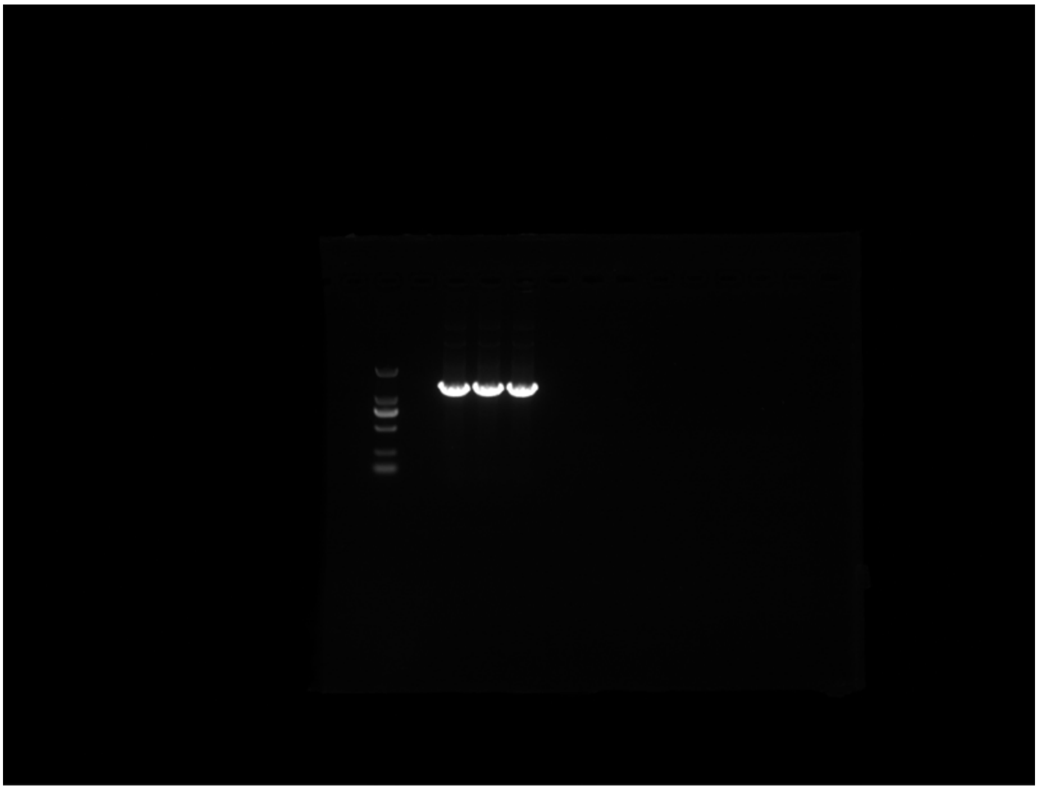

B: The raw gel of Supplementary Figure S1. B

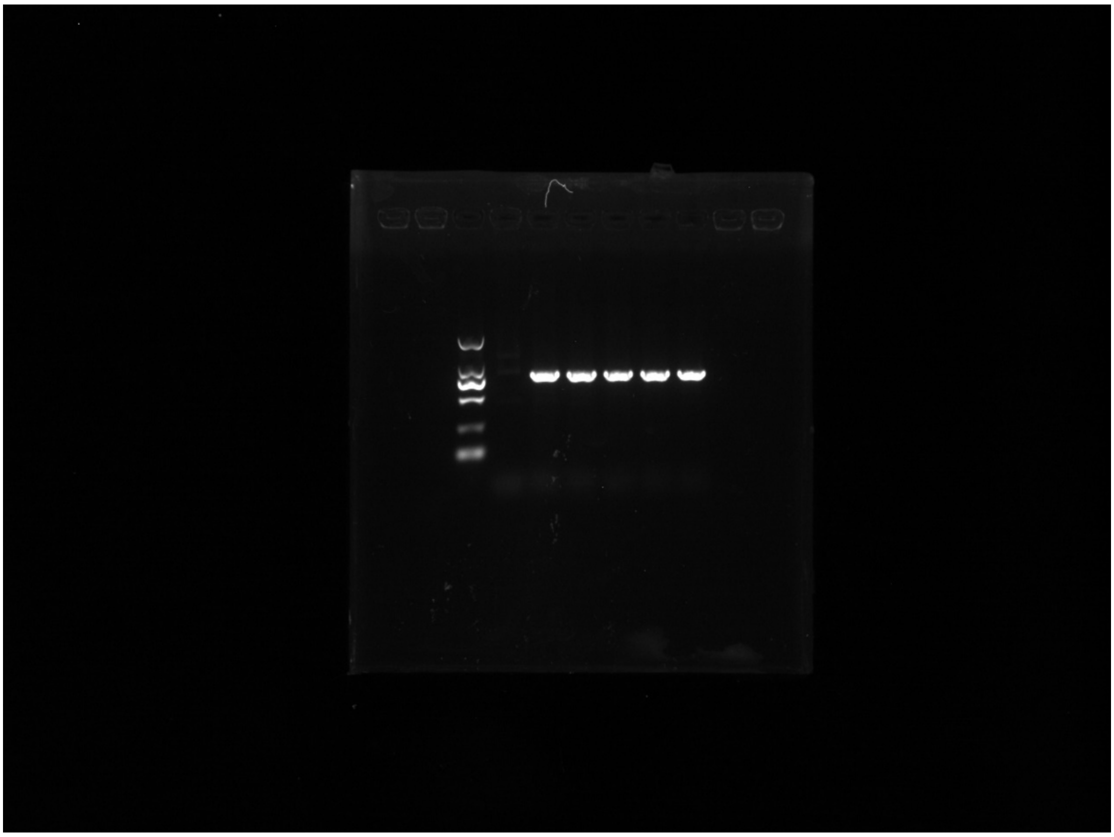

C: The raw gel of Supplementary Figure S1. C

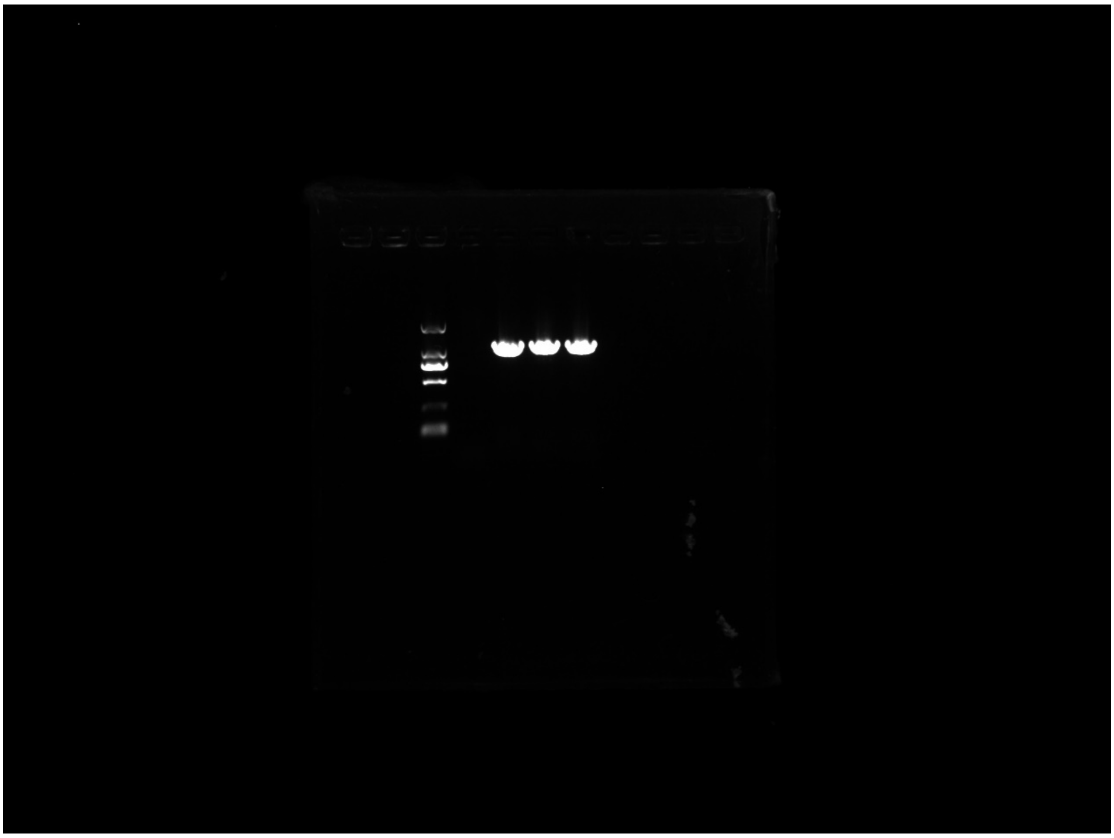

D: The raw gel of Supplementary Figure S1. D
